# Visual mismatch negativity elicited by facial expressions: new evidence from the equiprobable paradigm

**DOI:** 10.1186/1744-9081-8-7

**Published:** 2012-02-02

**Authors:** Xiying Li, Yongli Lu, Gang Sun, Lei Gao, Lun Zhao

**Affiliations:** 1 Institute of Developmental Psychology, Beijing Normal University, Beijing, China; 2 College of Education Science, Henan Normal University, Xinxiang, China; 3 School of Psychology, Beijing Normal University, Beijing, China; 4 Department of Medical Imaging, Jinan Military General Hospital, Jinan, Shandong Province, China; 5 Center for Visual Art & Brain Cognition, Beijing Shengkun Yan Lun Technology Co., Ltd., Beijing, China

## Abstract

**Background:**

Converging evidence revealed that facial expressions are processed automatically. Recently, there is evidence that facial expressions might elicit the visual mismatch negativity (MMN), expression MMN (EMMN), reflecting that facial expression could be processed under non-attentional condition. In the present study, using a cross modality task we attempted to investigate whether there is a memory-comparison-based EMMN.

**Methods:**

12 normal adults were instructed to simultaneously listen to a story and pay attention to a non-patterned white circle as a visual target interspersed among face stimuli. In the oddball block, the sad face was the deviant with a probability of 20% and the neutral face was the standard with a probability of 80%; in the control block, the identical sad face was presented with other four kinds of face stimuli with equal probability (20% for each). Electroencephalogram (EEG) was continuously recorded and ERPs (event-related potentials) in response to each kind of face stimuli were obtained. Oddball-EMMN in the oddball block was obtained by subtracting the ERPs elicited by the neutral faces (standard) from those by the sad faces (deviant), while controlled-EMMN was obtained by subtracting the ERPs elicited by the sad faces in the control block from those by the sad faces in the oddball block. Both EMMNs were measured and analyzed by ANOVAs (Analysis of Variance) with repeated measurements. sLORETA (standardized low-resolution brain electromagnetic tomography) was used to investigate the cortical generators of controlled-EMMN.

**Results:**

Both the oddball-EMMN in deviant-standard comparison and the controlled-EMMN in deviant-control comparison were observed at occipital-temporal regions with right hemisphere predominance. The oddball-EMMN was bigger and earlier than the controlled-EMMN because, besides the memory-based comparison, the former included a difference of refractoriness due to the distinction of presented probability between the deviant and standard face stimuli. The source analysis of controlled-EMMN indicated a current source primarily involved in posterior areas including superior temporal gyrus, postcentral gyrus, inferior parietal lobule as well as the insula.

**Conclusions:**

The valid EMMN properly reflecting the memory-based comparison of facial expressions could be obtained, i.e., the controlled-EMMN.

## Background

Faces are ecologically important stimuli that provide essential cues that form the basis for interpersonal communication. In particular, emotional expressions of faces can convey information with regard to a person's mental states, intentions, or dispositions. Therefore, it is not surprising that considerable efforts have been invested to investigate how the brain processes facial expressions. Converging evidence revealed that facial expressions are processed automatically (see [1] for a review). Using the ERPs (event-related potentials) method, it was found that the facial expression was encoded within the first 300 milliseconds after the appearance of the stimulus [2-4]. Recently, there is evidence that facial expressions might elicit a visual mismatch negativity (MMN), expression MMN (EMMN), reflecting that facial expressions could be processed under non-attentional condition [5].

The mismatch negativity (MMN) component reflects the differences between the ERPs elicited by deviant (infrequent) and standard (frequent) stimuli and it is related to pre-attentive memory-based comparison [6,7]. While being well defined in the auditory modality, recent studies provided fairly convincing evidence for the existence of the visual MMN (see [8] for a review), such as color [9], motion direction [10,11], orientation [12,13], spatial frequency [14], luminance [15], size [16]. The visual mismatch negativity (vMMN) is described as a negativity measured at the temporo-occipital electrodes with variable latency between 150 and 350 ms post the stimulus onset. Although a few works showed that vMMN may be based on rareness of events [9,17], the vMMN with a posterior scalp distribution reflecting the memory-based detection of deviant visual stimuli has been exhibited especially when stimulus-specific refractoriness as a major factor in the generation of traditional vMMN was ruled out [8,12,13,18-20].

In addition to vMMN elicited by changes of low-level simple visual features, particularly relevant to the present study, several studies investigated the possibility that complex visual information such as facial expressions might elicit a vMMN. Using a modified "cross-modal delayed response" paradigm, Zhao and Li [5] first reported an expression-related mismatch negativity (EMMN, neutral faces as standard stimuli) at the latency of 110-430 ms with a right-posterior scalp distribution. Unfortunately, the face pictures of one person only were used in their study and hence, the EMMN could be not based on emotional content of faces but change detection of the low-level visual information *per se*. To eliminate the low-level effects, using pictures with varying facial identity in oddball paradigm one recent study found that compared to neutral expressions (standard stimuli), the deviant (fearful and happy) expressions elicited the occipital negativity at the latency of 150-180 ms [21]. Most recently, to minimize the variance associated with human facial photographs as stimuli, using schematic sad and happy faces as deviants and schematic neutral faces as standard stimuli Chang et al [22] found the EMMN similar to Zhao and Li's finding [5]. It is noteworthy that in the above studies, the EMMN was obtained by subtracting ERP waveforms in response to frequent standard stimuli from those in response to infrequent deviant stimuli. It has been shown that stimulus repetition leads to repeated initiation of patterns of neural activity that habituates as a function of repetition rate [23]. These refractory effects can suppress the neural response to standard stimuli in the oddball sequence and importantly, this suppression is greater for standard stimuli than for the infrequent deviant stimuli. Thus, the brain could detect changes in facial expressions based simply on the basis of differential states of refractoriness of neurons specifically responding to given frequencies [8,24-27]. Hence, it is necessary to further investigate the existence of a memory-comparison-based EMMN.

To address the above question, in the present study, we will employ the equal probable paradigm utilized by Jacobsen and Schröger [26,27] to verify memory-comparison-based explanations of MMN, which has been used in visual modality [12,13,18]. Instead of traditional comparison of ERPs elicited by infrequent deviant and frequent stimuli, the ERP waveforms to deviant stimuli in the oddball sequence block are compared with those to the same physical stimuli in control sequence block presenting several different stimuli whose probability is equal to that of the deviant in the oddball sequence. The rationale behind this method is that the stimulus-specific refractoriness of deviant stimuli is approximately equal in the two types of sequences and that in the control sequence none of stimuli breaks the regularity. Therefore, the MMN will be elicited only in the oddball sequence and calculated as the difference waveforms between ERPs elicited by deviant stimuli in oddball sequence and those by same stimuli in control equiprobable sequence [12,13,18,24-27]. In the present study, the infrequently occurring stimuli (sad faces) in the oddball sequence block are deviant stimuli and the neutral faces are standards, and in the control sequence block, the identical sad faces are presented with other four kinds of face stimuli with equal probability as in the oddball sequence. If there is a purely memory-comparison-based EMMN, it could be obtained by subtracting the ERPs elicited by sad faces in the control block from those by identical sad faces (deviant stimuli) presented in the oddball block. In addition, if the face-specific N170 is contributed to expression MMN as previous studies [5,21,22] the sad faces in oddball block would elicit lager N170 than did those in control block although they are physically the same.

## Methods

### Participants

The participants were 12 undergraduates (6 female, 20-23 years old) from Xuzhou Normal University in China. All participants had normal or corrected to normal visual acuity and had no history of psychiatric or neurological disorders. They were right handed based on self report and paid for participation.

### Stimuli and procedure

**Figure 1:**
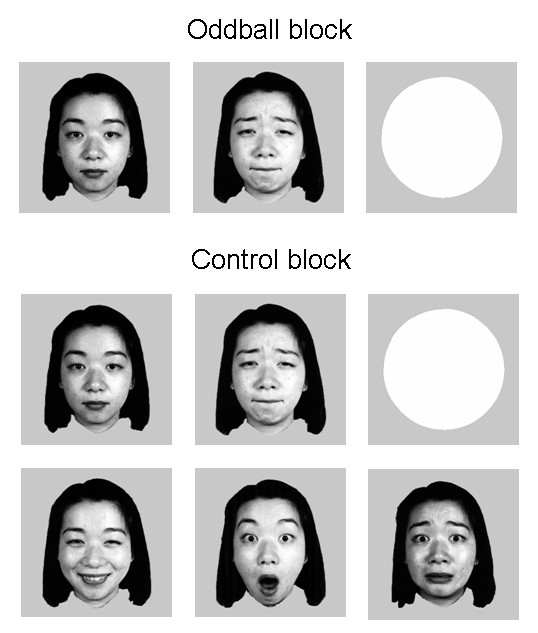
**The standard (neural face) and deviant (sad) faces in oddball block and faces with five kinds of expressions in control block as well as the target stimulus (non-patterned white circle)**.

Similar to the method proposed by Maekawa et al [28], subjects were instructed to focus their attention on a story delivered binaurally through earphones, while looking at the center of the monitor. Half of the subjects were asked to press a button with their right thumb as soon as they recognize a target stimulus, i.e., a non-patterned white circle, on the screen, and the other half responded with their left thumb. The faces as non-target visual stimuli were B&W photographs of five Asian women selected from the Japanese Female Facial Expression (JAFFE) database [29], wearing either neutral, sad, surprise, fear, or happy expression (Figure 1). All the visual stimuli were presented for 200 ms at the center of monitor, with a visual angle of 2.58° × 2.4°. After stimuli run, subjects were instructed to fill out a questionnaire consisted of 25 questions about the context of the story that they have heard.

According to the method proposed by Schröger and Wolff [24] in the auditory modality, which has been used in the visual modality [12,13,18], there were two blocked conditions in the current experiment: (a) deviant sad face with standard neutral face (oddball block); (b) control comprised of all five faces with equal probability as deviant faces in the oddball block (control block). For each blocked condition there were two sequences with 20 targets and 180 faces for each, that is, 72 sad faces and 288 neutral faces in the oddball block and 72 stimuli for each facial expression in the control block. The inter-trials interval ranged randomly between 800 ms and 1200 ms. The order of blocked conditions were counterbalanced across subjects.

#### EEG record

Electroencephalogram (EEG) was continuously recorded (band pass 0.05-100 Hz, sampling rate 500 Hz) with Neuroscan Synamp^2 ^Amplifier, using an electrode cap with 64 Ag/AgCl electrodes mounted according to the extended international 10-20 system and referenced to the tip of the nose. VEOG and HEOG were recorded with two pairs of electrodes, one placed above and below right eye, and the other 10 mm from the lateral canthi. Electrode impedance was maintained below 5 kΩ throughout the experiment.

EOG artifacts were corrected using the method proposed by Semlitsch et al [30]. The EEG was segmented in epochs of 1000 ms, time-locked to faces onset and included a 200 ms pre-stimulus baseline. Trials contaminated by amplifier clipping, bursts of electromyographic activity, or peak-to-peak deflection exceeding ± 100 μv were excluded from averaging. The EEG segments were averaged separately for neutral and sad faces in both blocks. The average number was 62.3, 259.5, and 60.7 for sad, neutral faces in oddball block and sad faces in control block, respectively. The averaged ERPs were digitally filtered with a low-pass filter at 30 Hz (24dB/Octave).

Two kinds of "Expression MMN" (EMMN) were calculated: (a) oddball-EMMN in the oddball block by subtracting the ERPs elicited by the standard stimuli (neutral) from the deviant stimuli (sad); (b) controlled-EMMN by subtracting the ERPs elicited by the sad stimuli in the control block from the identical stimuli (i.e., deviant sad faces) in the oddball block.

The statistical analysis was based on within-subject factorial models in which the amplitudes of original ERP components (temporo-occipital N170 and P2 components) and subtraction-derived EMMN were dependent variables. The measurement windows were determined by visual inspection of grand average waveforms, 110-210 ms and 180-300 ms for the peak amplitudes and latency of N170 and P2, respectively, and three 100 ms time windows for EMMN (110-210 ms, 210-310 ms and 310-410 ms). Peak and mean amplitudes were assessed via ANOVAs with repeated measurements. The degrees of freedom were corrected using the Greenhouse-Geisser epsilon.

#### Source analysis of EMMN

The computation of images of electric neuronal activity based on extracranial measurements would provide important information on the time course and localization of brain function. In the present study, we attempted to analyze the cortex source of EMMN by an academic software, "sLORETA", in which the sLORETA method (standardized low-resolution brain electromagnetic tomography) was run with a standardized boundary element method volume conductor model [31,32] and the expanded electrode coordinate (MNI, Montreal Neurological Institute stereotactic coordinates) showed a validity as relative head-surface-based positioning systems [33], and it has been shown that sLORETA can yield images of standardized current density with zero localization error [34]. sLORETA-images for ERPs to the sad faces in oddball and control conditions were compared with a voxel-by-voxel t-test. The multiple comparisons were corrected by a randomized test based on statistical non-parametric mapping (SnPM, number of randomizations: 5000) [35]. The voxels with significant differences (p < 0.05) were projected in specific brain regions.

## Results

### Behavioral data

In order to know the degree of attention, accuracy of answers to questions on the story as well as the reaction time (RT) and accuracy of target stimuli were evaluated. Mean correctness rate for questions related to the story was 98.2%, showing that subjects were paying attention to the story. In addition, mean RTs and accuracy of target stimuli had no significant effect of conditions (oddball and control blocks, *ps *> 0.1), 380 ms with an accuracy of 98.65% and 378 ms with 98.95% accuracy, respectively.

### ERPs data

**Figure 2:**
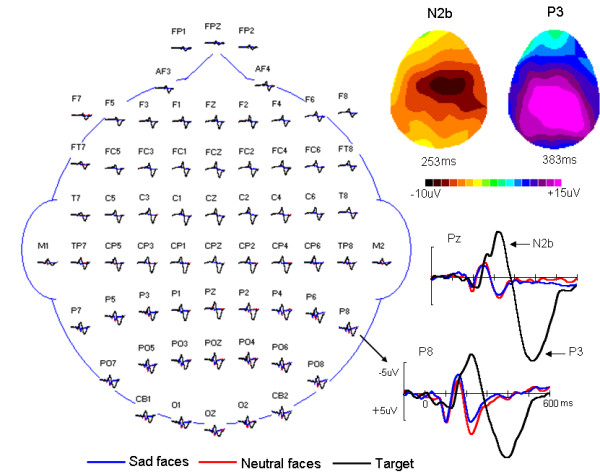
**Topographic arrangement showing grand averages of ERPs in response to standard (red), deviant (blue) and target (black) stimuli under the oddball condition**. Clearly, the N2b-P3 complex with frontal (N2b)/parietal (P3) distribution was elicited only by target stimuli.

Grand average of ERPs in response to standard (neutral faces), deviant (sad faces) and target stimuli (white circle) under oddball block condition were shown in Figure 2. At posterior electrode sites (e.g., P8), the P1-N1/N170-P2 deflection was elicited by standard and deviant stimuli. However, the N2b-P3 complex only appeared with the target stimuli with the maximum at Fz (N2b component) and Pz (P3 component) sites. On the basis of the aim of the present study, we focus on the analysis of ERPs in response to face stimuli, that is, standard neutral faces and deviant sad faces in oddball-block condition as well as sad faces in control-block condition.

### N170 and P2 components

**Figure 3:**
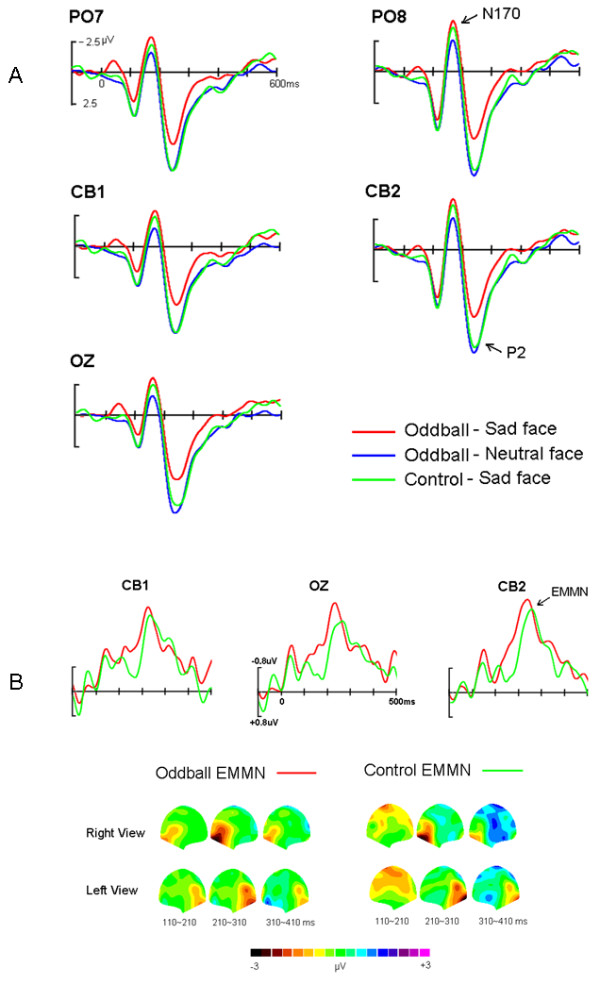
**(A) The grand-average ERPs elicited by standard (neutral faces) and deviant (sad faces) in oddball block and sad faces in control block, respectively**. (B) The grand-average oddball-EMMN (expression mismatch negativity) and controlled-EMMN and the 2D scalp topographic distributions of two EMMNs.

Figure 3(A) showed the grand-average ERPs elicited by standard (neutral faces) and deviant (sad faces) in oddball block and sad faces in control block, respectively. All face stimuli elicited N170 and P2 components. In two comparisons (deviant vs. standard, deviant vs. control), three-way ANOVAs of the peak amplitudes and latencies of N170 and P2 were calculated with factors of Stimulus Type (deviant sad and standard neutral faces, deviant sad and control sad faces, respectively), Hemisphere (left and right) and Site (P7/P8, PO7/PO8, CB1/CB2, O1/O2). First, for deviant vs. standard comparison, the main effects of Stimulus Type for peak amplitudes of N170 and P2 were significant, *F*(1, 11) = 6.58, *p <*0.03 and *F*(1, 11) = 5.70, *p <*0.05, respectively, reflecting that sad faces elicited larger N170 (-3.8 μV) and smaller P2 (5.9 μV) than did neutral faces (-2.7 μV, 8.0 μV). Additionally, both N170 and P2 showed a significant main effect of Site, *F*(3, 33) = 5.07, *p = *0.03, and *F*(3, 33) = 5.45, *p <*0.03, respectively, with the amplitude biggest at PO7/8 (-3.9 μV) for N170 and more occipital distribution (O1/2, 7.7 μV) for P2. On the other hand, for deviant vs. control comparison, the amplitude of N170 was almost identical (-3.8 μV vs. -4. 0 μV, *F*(1, 11) < 1), whereas the P2 was significantly decreased for deviant sad (5.9 μV) than control sad faces (7.6 μV, *F*(1, 11) = 6.15, *p <*0.035). Similar to the deviant vs. standard comparison, significant Site effects for peak amplitudes of the N170, *F*(3, 33) = 5.22, *p = *0.030, and P2, *F*(3, 33) = 7.27, *p <*0.01, were found. In addition, for the latencies of N170 and P2, neither main effects nor interactions were significant for both comparisons.

### Oddball- and controlled-EMMN

Figure 3(B) presented the grand-average oddball-EMMN and controlled-EMMN, peaked at 230 ms and 250 ms post stimuli, respectively. Clearly, oddball-EMMN was more negative than controlled-EMMN in the time window of early stage of EMMN. Based on the scalp topographic distribution of EMMN, it is conspicuous that both EMMNs were distributed at posterior areas: occipito-temporal areas (e.g., PO8, CB2), and covered larger areas in the right than in the left hemisphere sites. The statistical reliability of the above pattern was tested by a three-factor ANOVA of the EMMN mean amplitudes (110-210 ms, 210-310 ms, and 310-410 ms, respectively), with EMMN Type (oddball-EMMN and controlled-EMMN), Hemisphere (left and right) and Site (O1/O2, PO7/PO8 and CB1/CB2) as within-subject factors. In the 110-210 ms range, the main effects of EMMN Type and Site were significant, *F*(1, 11) = 7.53, *p <*0.02, and *F*(2, 22) = 6.04, *p <*0.035, respectively, reflecting that oddball-EMMN was more negative (-1.1 μV) than controlled-EMMN (-0.5 μV) and that the EMMN amplitude was largest (-0.9 μV) at CB1/2 site. No other main effects and interactions reached significant level. In the other two time ranges, no main effect of EMMN Type was found. However, in the second time window (210-310 ms), there was a significant main effect of Hemisphere, *F*(1, 11) = 8.50, *p <*0.02, showing a right occipito-temporal hemisphere predominance of EMMN.

### Source analysis of the controlled-EMMN

**Figure 4:**
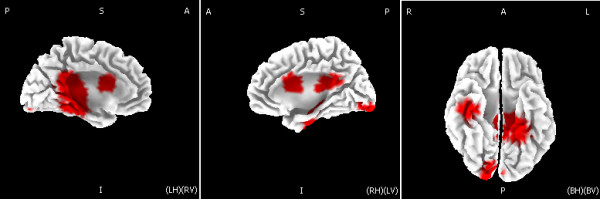
**Graphical representations of the sLORETA results comparing the amplitudes of ERPs elicited by the deviant-sad and control-sad face stimuli**. Areas colored in red represent the place where the significant differences between the deviant and control activities (p < .05) were shown.

Figure 4 showed current sources of the control EMMN in the time window between 215 and 285 ms post stimuli onset, as estimated by using sLORETA. Current sources of EMMN were located in the insula (BA 13, sub-lobar), superior temporal gyrus (BA 41, temporal lobe), postcentral gyrus (BA 2, parietal lobe), inferior parietal lobule (BA 40, parietal lobe) as well as cingulate gyrus (BA 31, limbic lobe), with a current density maximum of 4.88 μAmm/mm^2 ^at the location X = -30, Y = -30, Z = 20 mm (the insula), in MNI stereotactic coordinates.

## Discussion

Using the cross-modality paradigm, in the present study we investigated pre-attentive processing of face expressions by recording the expression-related visual mismatch negativity (expression MMN, EMMN). To observe genuine memory-comparison-based EMMN, stimulus-specific refractoriness was eliminated by using equiprobable control block. As expected, two kinds of EMMNs, oddball-EMMN in deviant-standard comparison in oddball block and controlled-EMMN in deviant-control comparison, were found with posterior scalp distribution. In particular, the oddball-EMMN was bigger and earlier than the controlled-EMMN because the face-associated N170 did not contribute to the latter.

Similar to auditory MMN studies, a suitable experimental approach for visual MMN would involve maintaining the subject's attention to a continuous task in which the task stimuli are independent of irrelevant ones. In the present study we adopted the cross-modality method proposed by Maekawa et al [28], in which subjects were instructed to focus their attention on a story delivered binaurally through earphones, while looking at the center of the monitor, and press a button as soon as they recognize a visual target stimulus. The visual target task could make subject to really look at the center of monitor so that the visual MMN could be elicited by no-target stimuli. Obviously, it is possible that subjects, indeed, attend the visual modality. However, in Maekawa et al' study [28], the authors found that only when irrelevant deviants were close resemblance to the target they did capture the attention of the subject, reflecting by the fact that deviants interfered with target in terms of the RT results. In contrast, irrelevant deviants did not interfere with target identification when they were very different from the target. Similarly, in the present study, the visual target stimulus (i.e., white circle) was very different from non-target standard and deviant stimuli and the subjects were asked to attention the auditory story throughout the experiments. In addition, the attention specific N2b-P3 complex [36] was elicited only by visual target stimuli but not by deviant faces (see Figure 2). As proposed by Maekawa et al [28], therefore, these results support the idea that subject's attention was almost shifted away from the non-target deviant stimuli. In consistent with our previous study [5,22], compared with the ERP waveform of standard neutral faces as well as control sad faces, a clear negative shift over posterior scalp was elicited by deviant sad faces, regardless of control and oddball conditions, that is, a valid EMMN was observed. We believed that the EMMN reported here reflected pre-attentive expressional information processing.

Compared with standard neutral faces in oddball block, infrequent sad faces elicited posterior negativities between 100 ms and 350 ms, which is highly consistent with previous facial expression oddball study [5,22]. The posterior negativity observed in the deviant-versus-standard comparison, i.e., oddball-EMMN, indeed consisted of two subsequent posterior negativities, early negativity (100-200 ms) based on N170 difference and late negativity (200-350 ms) due to P2 difference. In line with the present N170 effect in oddball sequence, using similar cross-modality oddball paradigm one recent study showed that deviant expressional faces elicited larger N170 than frequent neutral faces, resulting in the occipital negativity at the latency of 150-180 ms, and the authors considered that the N170 could be suitable counterpart of visual EMMN [21]. However, some visual MMN studies indicated that the traditional MMN in the oddball sequence indeed confounds standard stimuli refractoriness reflected by the changes of early visual ERP components such as the temporo-occipital N1 component (N170 component in the present study) [13,18]. Recently, Kimura et al. [13] investigated directly the underlying processes of the visual MMN and considered that the early posterior negativity peaked at around 100-150 ms reflects visual N1 refractory effect, while the late negativity peaked at around 200-250 ms reflects memory-comparison-based change detection effect (that is, visual MMN). Indeed, the present EMMN time-course and distribution was also (partly) similar to the Kimura et al' vMMN study [13], e.g., occipital, right-hemispheric preponderant distribution, latency in the N1 to P2 range. Therefore, in the present study as well as previous EMMN studies the early part of oddball-EMMN based on N170 component changes could reflect stimulus-specific refractoriness. Supporting this notion, in deviant-versus-control comparison the N170 was not modulated at all and the controlled-EMMN was evident between 200-350 ms post stimuli onset. In the present experiment, control stimuli should not active change-specific neural populations and the state of refractoriness for control stimuli should be similar. Thus, the controlled-EMMN eliminated absolutely N170 refractory effect and reflects the memory-comparison-based change detection effect (i.e., genuine EMMN). At the same time, our data further support the suggestion that classical visual MMN in deviant-versus-standard comparison contains a contribution associated with neuron populations in different states of refractoriness responding to the standard and deviant stimuli [13,17].

Interestingly, in contrast to the present N170 effect on expression processing in deviant-versus-control condition, there is evidence that the face-associated N170 component was not totally insensitive to the facial emotional content even under non-consciousness condition (see [37] for a review). In those studies the comparison among different facial expressions was performed, while in the present deviant-control comparison the N170s elicited by expression stimuli with same physical features were compared. Since the N170 reflects an early activation of a domain-specific mechanism for visual analysis of faces [38], the present results suggest that pre-attentive memory-comparison-based processing of facial expressions occur after the perceptual processing relevant to the N170.

The topography analysis of the controlled-EMMN showed the occipito-temporal distribution (larger at right than at left hemisphere sites). In line with the present hemispheric specialization for change detection of sad expression, several recent EMMN studies showed that automatic processing of negative affect (e.g., fearful, angry) was associated with right hemisphere, while processing of positive affect (e.g., happy) evoked larger MMN in the left hemisphere [[5,22,39,40], but see [41]]. In addition, the source analysis of controlled-EMMN indicated a current source primarily involved in posterior areas including superior temporal gyrus, postcentral gyrus, inferior parietal lobule as well as the insula. Using sLORETA method like the present study, one recent source study of visual MMN elicited by orientation changes found that current sources of the visual N1 reflecting refractoriness effect were located in the occipital lobe (BA 17-19) and the visual MMN reflecting memory-comparison-based processing involved in neural activations of the occipital lobe (BA 19) and the frontal lobe (BA 47 and BA 11) [35]. Our results extend the vMMN study for simple visual features (e.g., color) to for complex visual information (i.e., facial expressions). The distinction between generators for expression MMN and visual MMN might indicate the specificity for visual MMN elicited by expression changes over by simple visual feature changes and further within-group studies are necessary to determine the distinction between vMMN and EMMN.

Particular relevant to present study, Kimura et al [40] found that for both fearful and happy faces, the neural generators of EMMN were located in temporal, occipital, limbic and frontal lobes. Obviously, there were partly diverging between Kimura et al and our present findings, in particular, we did not find the fontal activations of EMMN. In Kimura et al's study [40], the violated alternating pattern (i.e., perceptual learning pattern) was adopted to investigate the prediction error responses. Converging evidence indicated that prefrontal area plays an important role in error processing [42] and hence, it is possible that the EMMN generator is located in frontal lobe like in Kimura et al's study [40]. However, the EMMN in the present experiment was elicited in a simple random pattern (i.e., oddball sequence) that is related to sensory memory-based comparison not to repeating prediction processing. On the other hand, we did not use the fearful and happy expressions in the present study. Although converging evidence from functional imaging studies suggested that there is certain distinction of neural correlates for processing of different emotional facial expression, only a few studies explored the neural response to sad expressions [2]. For example, using a sex discrimination task, Blair et al [43] found that increasing intensity of sad facial expression was associated with enhanced activity in the left amygdala and right temporal region. It has been widely accepted that the areas including amygdale, cingulate cortex, and basal ganglia were activated during the sad expression recognition [44]. Interestingly, the present current sources of EMMN elicited by sad expression located in the insula with a maximum of current density. In contrast to this finding, insular activation has been selectively reported during processing of disgusted and angry faces in brain imaging studies [45]. As our knowledge, however, there were no brain-imaging studies about processing sad faces under the pre-attentive memory-based condition. Considering the methodological distinction between ERPs and functional imaging as well as the variety of valence and intensity of facial expressions, the neuro basis of processing sad faces under non-attentional condition awaits further investigation.

## Limitations

The present study has two limitations. First, although the behavioral and ERP data could imply that the deviant face stimuli did not capture subjects' attention, the task was to listen to a story and press a button to the visual target and hence, the possibility could not be ruled out completely that the subjects occasionally pay attention to the emotional state of the faces. Second, we performed this study with a limited number of participants. To generalize our results, further studies should be conducted involving a larger number of participants.

## Conclusions

In previous studies, the expression-MMN (EMMN) in deviant-versus-standard comparison contains a contribution associated with neuron populations in different states of refractoriness responding to the frequent standard and infrequent deviant facial expressions. The present study provides new evidence that facial expression can elicit the pre-attention memory-comparison-based EMMN.

## Competing interests

The authors declare that they have no competing interests.

## Authors' contributions

YL and LZ contributed to the design and planning of the experiment and cognitive tests, data analyses and writing the manuscript. XL took part in the planning and designing of the experiment and cognitive tests, data analyses and manuscript preparation. GS and LG contributed to the design and planning of the experiment. All authors read and approved the final manuscript.
